# Repair of atrioventricular disruption after mitral valve replacement utilizing commando procedure principles

**DOI:** 10.1093/icvts/ivac179

**Published:** 2022-07-01

**Authors:** Evgeny Raevsky, Lloyd Kwanten, Seamus Cullen, Serban Stoica

**Affiliations:** Department of Cardiac Surgery, St Bartholomew’s Hospital, London, UK; Department of Cardiac Surgery, St Bartholomew’s Hospital, London, UK; Department of Cardiac Surgery, St Bartholomew’s Hospital, London, UK; Department of Cardiac Surgery, St Bartholomew’s Hospital, London, UK; The Heart Institute, Bristol, UK

**Keywords:** Atrioventricular dissociation, Commando procedure, Mitral valve replacement, Congenital

## Abstract

Atrioventricular rupture is a life-threatening complication of mitral valve replacement. We present how incising the intervalvular fibrosa critically improves exposure. The aortic valve sacrifice allows access to a large atrioventricular dissociation defect and reliable repair of the anterolateral aspect of mitral valve annulus.

## INTRODUCTION

Atrioventricular (AV) groove disruption is caused by surgical technique or by stretch injury due to removal of posterior leaflet and untethering of left ventricle. It appears in about 1.2% of mitral replacements and the mortality rate is up to 75%. There is no easy technical solution, both external and internal approaches to repairing the disruption having been described [1]. The latter are preferred but reliable exposure can be problematic.

Aorto-mitral continuity reconstruction is typically employed when the intervalvular fibrosa is affected by infection. The ‘commando procedure’ is well described in endocarditis for reconstructing the fibrous skeleton [2]. We used the procedure previously in elective aorto-mitral enlargement for bi-valve upsizing [3]. Here, we describe its adaptation to an emergency situation.

## CASE REPORT

A 23-year-old female with Shone syndrome had coarctation repair as a child and a previous surgical valvotomy on a bicuspid aortic valve. She was referred for mitral valve replacement.

Preoperative echocardiogram showed severe mitral regurgitation and moderate stenosis via a funnel-shaped valve with parachute attachments to the posteromedial papillary muscle; the bicuspid aortic valve had mild regurgitation. Magnetic resonance imaging showed a dysplastic mitral valve, elongation of the anterior leaflet and tethering. Abnormal left ventricular myocardial architecture with false tendons and myocardial thinning were also noted.

She underwent elective redo mitral valve replacement via a transseptal/supra-septal approach. The posterior leaflet could not be preserved because of the funnel shape of the valve; furthermore, the abnormal subvalvar architecture would interfere with the leaflets of the mechanical prosthesis. A 29-mm bileaflet prosthesis was inserted. After coming of bypass uneventfully and protamine administration, the AV groove rupture gradually became apparent. The mitral valve prosthesis was removed through the same access. There was extensive AV groove dissociation extending to the anterolateral aspect of the annulus into the ejection pathway below the aortic valve, with friable and ill-defined margins. The conventional transatrial exposure of the defect was suboptimal, it was not possible to achieve a reliable internal repair with this access.

**Figure 1:**
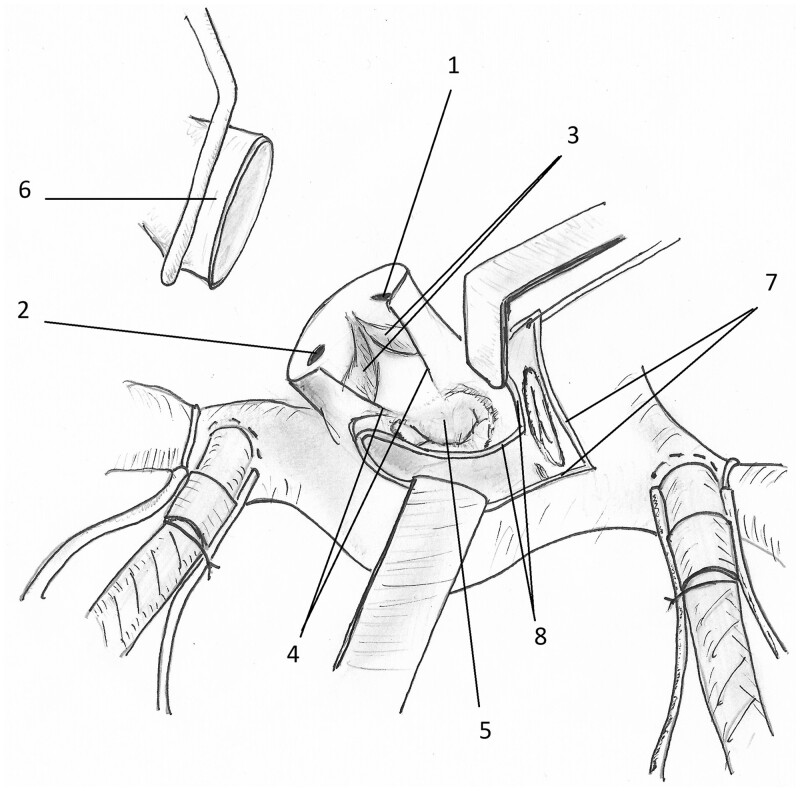
Vertical incision through the left/non-coronary commissure into the anterior mitral valve leaflet. (1) Right coronary ostium. (2) Left coronary ostium. (3) Left and right coronary cusps of the aortic valve. (4) Vertical incision through the non-coronary commissure towards the anterior mitral valve leaflet. (5) Mitral valve. (6) Distal part of the transsected ascending aorta. (7) Right atriotomy. (8) Atrial septal incision.

We employed a ‘commando technique’ to expose the anterolateral aspect of the subvalvar rupture. The aortic valve had to be sacrificed. The aorta was transected so as to tilt the base of the heart forward and improve exposure. The aortic root was then incised vertically through the non-coronary sinus and aorto-mitral curtain (Fig. 1). The transatrial access was extended towards that point and the heart was opened ‘like a book’. A full internal exposure of the AV groove defect was achieved. It was repaired using bovine pericardium and a 27-mm mechanical valve prosthesis position was attached to the patch posteriorly.

To recreate the aorto-mitral curtain and repair the inflow–outflow tracts, we used a vertical bovine pericardial patch, attaching the anterior part of the mitral prosthesis to its lower margin. A 21-mm mechanical valve was implanted in the native aortic annulus, halfway up along the vertical patch. The roof of left atrium was repaired with a separate horizontal patch. A Cabrol-type closure was used around the reconstructed root to control bleeding [4].

Antegrade cold blood cardioplegia was used every 20 min. The cumulative cardiopulmonary bypass and cross-clamp times were 475 and 310 min, respectively. The patient was discharged home after 50 days with moderate left ventricular dysfunction but no other major adverse outcomes.

## DISCUSSION

Age is a risk factor for AV groove rupture due to increased incidence of annular calcification and friable tissues. Our case shows, however, that this complication is also possible in a younger age group. Preservation of the posterior leaflet and basal chordae can help in prevention of AV rupture but is not always possible.

Whilst some forms of AV dissociation can potentially be handled via atriotomy, frank perforation and rupture carry high risk if not exposed adequately. This is partly due to the difficulty of surgical approach and proximity of circumflex artery.

**Figure 2:**
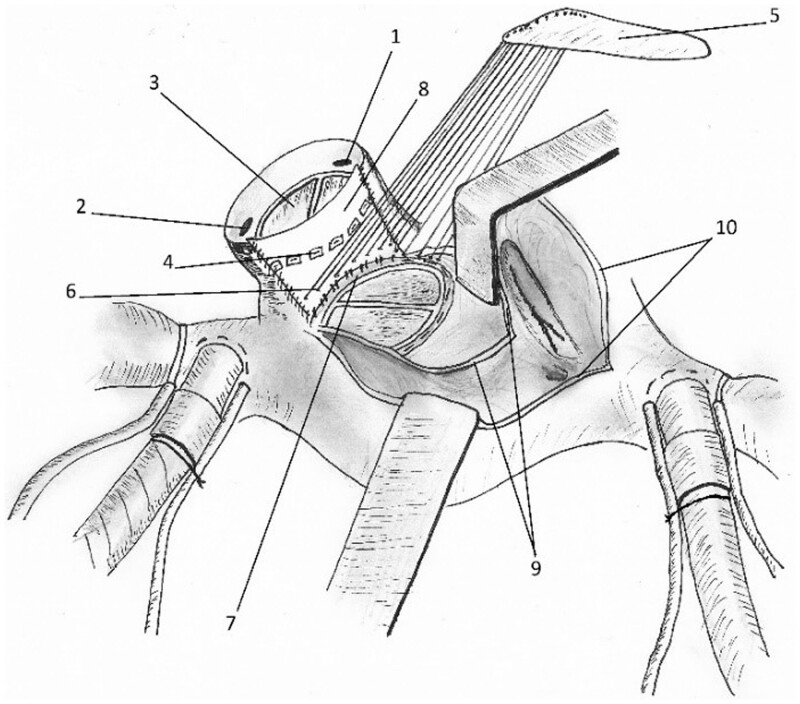
Completing the split-level reconstruction. (1) Right coronary ostium. (2) Left coronary ostium. (3) Aortic valve prosthesis. (4) Aortic prosthesis suture line. (5) Pericardial patch repairing roof of left atrium. (6) Suture line of left atrial patch inserting into atrioventricular curtain repair patch. (7) Mitral valve prosthesis suture inserting into atrioventricular repair patch posteriorly. (8) Aorto-mitral vertical repair patch. (9) Atrial septal incision. (10) Right atriotomy.

The commando procedure has potential outside endocarditis; we used it in children and young adults for bi-valve annular enlargement and the technical details are described elsewhere [3]. The aorto-mitral reconstruction patch carries suture lines from the mitral valve, patch of the roof of LA and aortic valve, respectively, in a split-level way (Fig. 2). Sacrificing the congenitally bicuspid aortic valve is not a major inconvenience in a patient committed to anticoagulation for the mitral valve replacement.

## CONCLUSION

AV groove rupture is frequently a lethal complication, which is best avoided by preventative measures. When it happens, there is no alternative to surgical repair. In this case of congenital mitral valve disease, the principles of the commando procedure allowed us to improve access and achieve reconstruction in an emergency situation unrelated to infection or elective double valve enlargement.

**Conflict of interest:** none declared.

## Data availability

No data are associated with this article.

## Reviewer information

Interactive CardioVascular and Thoracic Surgery thanks Ahmet Ruchan Akar and the other anonymous reviewer(s) for their contribution to the peer review process of this article.
